# Gone in a flash: manipulation of audiovisual temporal integration using transcranial magnetic stimulation

**DOI:** 10.3389/fpsyg.2013.00571

**Published:** 2013-09-11

**Authors:** Roy H. Hamilton, Martin Wiener, Daniel E. Drebing, H. Branch Coslett

**Affiliations:** ^1^Department of Neurology, University of PennsylvaniaPhiladelphia, PA, USA; ^2^Center for Cognitive Neuroscience, University of PennsylvaniaPhiladelphia, PA, USA; ^3^Department of Psychology, University of PennsylvaniaPhiladelphia, PA, USA; ^4^Moss Rehab Research InstituteElkins Park, PA, USA

**Keywords:** transcranial magnetic stimulation, noninvasive brain stimulation, sound-induced flash illusion, audiovisual integration, inferior parietal lobule (IPL), angular gyrus (AG)

## Abstract

While converging evidence implicates the right inferior parietal lobule in audiovisual integration, its role has not been fully elucidated by direct manipulation of cortical activity. Replicating and extending an experiment initially reported by Kamke et al. (2012), we employed the sound-induced flash illusion, in which a single visual flash, when accompanied by two auditory tones, is misperceived as multiple flashes (Wilson, 1987; Shams et al., 2000). Slow repetitive (1 Hz) TMS administered to the right angular gyrus, but not the right supramarginal gyrus, induced a transient decrease in the Peak Perceived Flashes (PPF), reflecting reduced susceptibility to the illusion. This finding independently confirms that perturbation of networks involved in multisensory integration can result in a more veridical representation of asynchronous auditory and visual events and that cross-modal integration is an active process in which the objective is the identification of a meaningful constellation of inputs, at times at the expense of accuracy.

## Introduction

Audiovisual integration is a critical feature of sensory processing that allows for the creation of coherent percepts from disparate sensory streams. Integration occurs readily when auditory and visual stimuli are coincident in space and time (Stein, 1998), but may also occur in the presence of incongruities (e.g., Templeton et al., 1966; Driver, 1996; Fendrich and Corballis, 2001). Resolution of such incongruities can give rise to perceptual illusions in which stimuli in one sensory domain affect perception in the other. Examples include the ventriloquist effect, in which visual stimuli affect perception of sound location, and the McGurk effect, in which incongruous lip movements alter the perception of speech sounds (McGurk and MacDonald, 1976; De Gelder and Bertelson, 2003).

The ability to perceptually conjoin synchronous and near-synchronous events from different modalities is of central importance to audiovisual integration. According to some recent models of temporal processing the right inferior parietal lobule (RIPL) may have a specific role in both unimodal and multimodal event order judgments (Snyder and Chatterjee, 2004; Battelli et al., 2007), and may contribute to the perception of synchrony between events across sensory modalities. Consistent with this and with some prior imaging results, we described a patient with right parietal injury who acquired an isolated inability to integrate synchronous auditory and visual events, perceiving simultaneous stimuli (e.g., spoken speech sounds and congruent lip movements) as being mismatched in time (Hamilton et al., 2006; see also Calvert, 2001; Bernstein et al., 2008).

Until recently, studies of audiovisual integration had not differentiated anatomical sites within the RIPL. Kamke et al. (2012) used transcranial magnetic stimulation (TMS) to fractionate two areas in the RIPL—the angular gyrus (AG) and the supramarginal gyrus (SMG)—in order to examine their roles in audiovisual integration. Employing a perceptual phenomenon known as the sound-induced flash illusion, wherein a single visual flash accompanied by two auditory beeps is often misperceived as multiple flashes (Wilson, 1987; Shams et al., 2000), Kamke and colleagues interrogated the two regions' roles in audiovisual integration by attempting to suppress illusory percepts using TMS. The group's results suggested that disruption of the angular gyrus, but not the supramarginal gyrus, influenced the rate of participants' perception of the illusion. We sought to replicate these findings by differentiating the functions of the angular and supramarginal gyri with respect to audiovisual integration.

## Materials and methods

### Participants

Nine right-handed subjects (7 females, 2 males; mean age 26 years) participated. All subjects gave informed consent as approved by the University Institutional Review Board and were naïve to the nature of the illusion.

### Task

**Figure 1:**
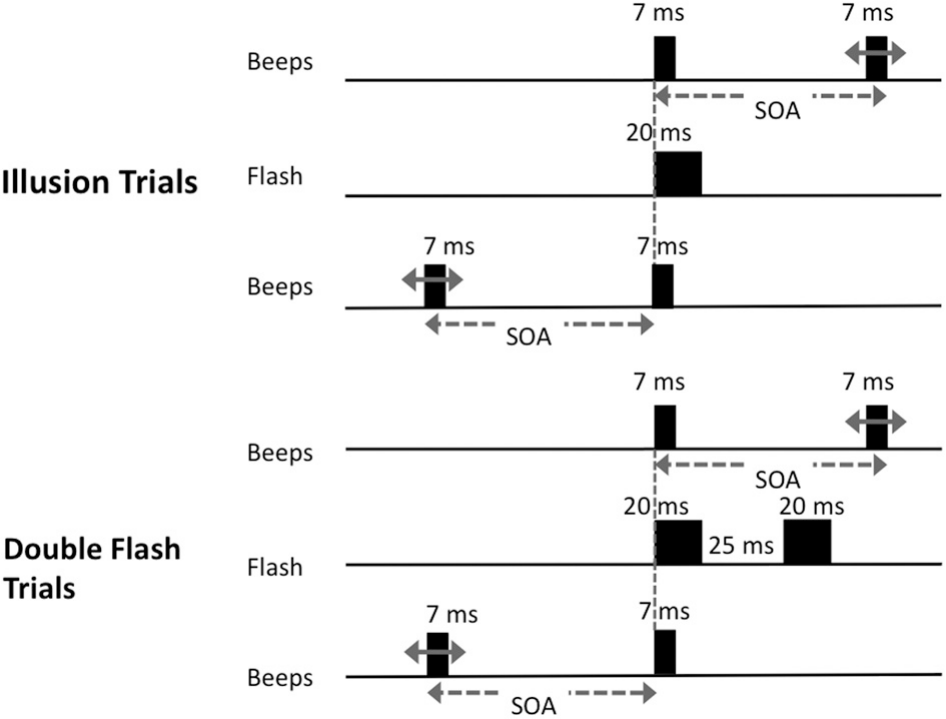
**Schematic of trial types presented**. On illusion trials, a single visual stimulus was flashed at the center of the screen along with pair of beeps. One beep was synchronous with the onset of the flash; the other occurred at one of 14 possible stimulus onset asynchronies (SOAs) before or after the flash (−250, −205, −160, −115, −70, −50, −25, 25, 50, 70, 115, 160, 205, or 250 ms); On double-flash trials, two flashes were presented with the same set of SOA values as in illusion trials.

Participants viewed stimuli displayed on a CRT monitor with a white background and a refresh rate of 100 Hz, connected to a laptop computer. Subjects were seated at a comfortable distance from the monitor. Auditory stimuli were presented via headphones. Prior to the beginning of testing, each subject adjusted the sound volume to a comfortable and easily audible level. At the beginning of each trial, the words “Begin Test” were displayed at the center of the screen for 2 s or until a keypress was detected. A fixation point was presented 6.5 cm from the top of the screen for 3 s. There were two trial types (Figure 1). On “illusion trials,” a solid black disc was flashed (20 ms) at the center of the screen and a pair of 7 ms beeps with a frequency of 3.5 kHz was presented. The beeps were generated using the Audacity 2.0 (http://audacity.sourceforge.net/) tone generator function, and were simple sine-wave sounds with no ramping in the sinusoids. There were 14 types of “illusion trials” that differed with respect to the interval between the two beeps (the stimulus onset asynchrony; SOA) and in which one of the two beeps was synchronous to the flash. The onset of one beep was always concurrent with the onset of the flash. On half of the illusion trials, the onset of the second tone coincided with the onset of the flash and the first tone preceded the beep/flash by 250, 205, 160, 115, 70, 50 or 25 ms; on other trials the first tone coincided with the onset of the flash and the second tone followed after one of the seven intervals noted above.

On “double-flash trials,” two 20 ms flashes were presented with a 25 ms inter-onset-interval. A pair of beeps was also presented with the same set of SOA values as in illusion trials. There were 14 types of double flash trials that differed with respect to SOA. On half of the trials, the first tone coincided with the first flash, while the second tone occurred 250, 205, 160, 115, 70, 50 or 25 ms later. On the other half of trials the first tone preceded the first flash by 250, 205, 160, 115, 70, 50 or 25 ms, and the second tone was simultaneous with the first flash. The interval between flashes was fixed at 25 ms because we sought to parallel the control conditions employed in earlier key investigations of this illusion (Shams et al., 2002). We also wished to avoid trials in which the double-flashes were presented at such long SOAs that they would be too easy to discriminate. Moreover, by interspersing double-flash trials as designed with single-flash trials, we prevented subjects from learning that there is always only one flash at short SOAs, and from adopting a conscious strategy of reporting single flash when beeps were presented at short intervals.

After each trial subjects indicated the number of flashes they perceived by pressing the corresponding number key on a USB keyboard positioned in front of them. Subjects were instructed to respond as quickly but as accurately as possible. Each session consisted of 140 trials in randomized order, with 5 trials at each SOA (positive and negative). The task duration was approximately 10 min. No feedback regarding accuracy was provided.

Our protocol differed from that of Kamke et al. (2012) in two specific ways, in order to further characterize the sound-induced flash illusion as well as any TMS-induced effects on that illusion. Whereas Kamke and colleagues presented illusion trials with only one flash and beep, followed by another beep at 70 or 160 ms post-stimulus onset, we presented illusion trials with beeps before and after flashes using a variety of SOAs; in doing so, we hoped to gain a clearer sense of the temporal window of intersensory stimulus presentation within which the illusion was most robust. We also included a two flash, two beep condition, which was not included in the Kamke paper, because we were interested in whether an illusory perception of additional flashes (i.e., >2) could be induced even when the number of flashes and beeps were matched.

### Experimental protocol: AG and SMG stimulation

Subjects first participated in a baseline session to determine if they perceived the illusion. Subjects completed the task three times to ensure stable performance; only data from the third run were analyzed. Subjects who reported seeing two or more flashes on 40% or greater of all illusion trials participated in two TMS sessions on separate days. Using this criterion, 12 individuals underwent baseline testing but were excluded from participating in TMS sessions. In the nine subjects who received TMS, the right AG and SMG were stimulated on different days and in random order between subjects. Subjects participated in the task twice during each TMS session. For 5 of the subjects, the task was administered before and immediately after receiving TMS. In the other 4 subjects, TMS was administered first, followed immediately by the task; subjects then waited 20 min before participating in the task a second time (“washout” period). This procedure was utilized to ensure that the effects of TMS on behavior were not simply due to practice or attentional effects resulting from subjects performing the task twice.

### Auditory control task

Nine additional subjects (3 males, 6 females; mean age 25 years) participated in an auditory control task in which no flashes were presented. Subjects were presented with either one 7 ms tone or two tones presented at SOAs of 25, 50, 70, 115, 160, 205, or 250 ms. There were 140 trials in total. In 70 trials only one tone was presented. In the other 70 trials, there were 10 trials at each of the seven SOAs. Subjects reported how many tones they had heard. This task was administered before and immediately after receiving TMS to the AG.

### Vertex control task

Subjects participated in a second control task in which stimulation of the midline Vertex was used. As described above for subjects undergoing AG and SMG stimulation, subjects were also screened, such that only those who reported seeing two or more flashes on 40% or greater of all illusion trials received TMS. Out of 15 subjects who underwent baseline testing, 6 were excluded and 9 (4 males, 5 females; mean age 26 years) received TMS. These subjects were presented with the same task as used for AG and SMG conditions. Similarly, for 5 of the subjects, the task was administered before and immediately after receiving TMS. In the other 4 subjects, TMS was administered first, followed immediately by the task; subjects then waited 20 min before participating in the task a second time.

### Brain stimulation

**Figure 2:**
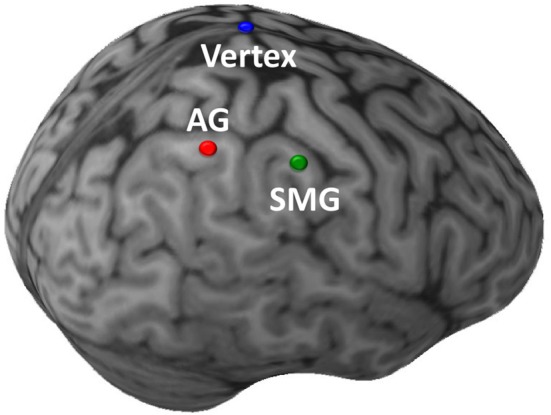
**Sites of TMS stimulation shown on the brain of a single illustrative subject**. The initial targets of stimulation were the anterior aspects of the right angular gyrus (AG; red) and supramarginal gyrus (SMG; green). In a subsequent control task, the target of stimulation was the Vertex (blue). (This image was created using Brainsight 2.0; Rogue Inc., Montreal, Canada.).

Stimulation was administered with a Magstim Rapid transcranial magnetic stimulator, connected to a 70 mm diameter figure-of-eight air-cooled coil (Magstim, Whitland, UK). The Brainsight system (Rogue Research, Montreal) was used to co-register data from a high resolution MRI of each subject's brain with the location of the subject and coil. Resting motor thresholds were determined using visual inspection. Consistent with prior work demonstrating that motor thresholds acquired using visual inspection can closely approximate those obtained using EMG (Pridmore et al., 1998), any perceptible movement of the thumb, wrist, or fingers was accepted as a motor response in our study. The anterior aspects of the right AG (approximate Talairach coordinates: 40, −63, 45) and SMG (52, −39, 41) were targeted (Figure 2). The coil handle pointed rearwards during vertex stimulation, and rearward and perpendicular to the orientation of the gyrus being stimulated for the angular gyrus or supramarginal gyrus sites. Repetitive TMS (rTMS) consisted of 1200 pulses administered with an intensity of 100% resting motor threshold at a frequency of 1Hz [~50μs pulse, 1-s inter-pulse-interval; for examples of similar methods see Walsh and Pascual-Leone (2005)]. An ~10–20 min duration of maximal TMS effect was anticipated, and the anticipated area of maximal effect at each cortical stimulation site was approximately ~1 cm^2^ (e.g., Chen et al., 1997; Touge et al., 2001; Wagner et al., 2007). All participants tolerated TMS well, and no adverse effects of stimulation were noted in any experimental sessions.

### Analysis

For each subject, the peak number of perceived flashes (PPF) during illusion and double flash trials was calculated across each of the 14 SOA trial types as the maximum mean number of flashes perceived in any SOA; the PPF measure thus represents a latency-independent measure of illusion strength. Based on the findings of prior studies (e.g., Shams et al., 2002). We noted that almost all subjects (8 of 9) perceived the sound-induced flash illusion maximally at SOAs ranging from −70 to +70 ms. (The ninth subject perceived the illusion maximally at −115 ms). This is consistent with prior work in which the illusion has been reported to be most robust in a range of −100 to +100 ms (Shams et al., 2002).

## Results

**Figure 3:**
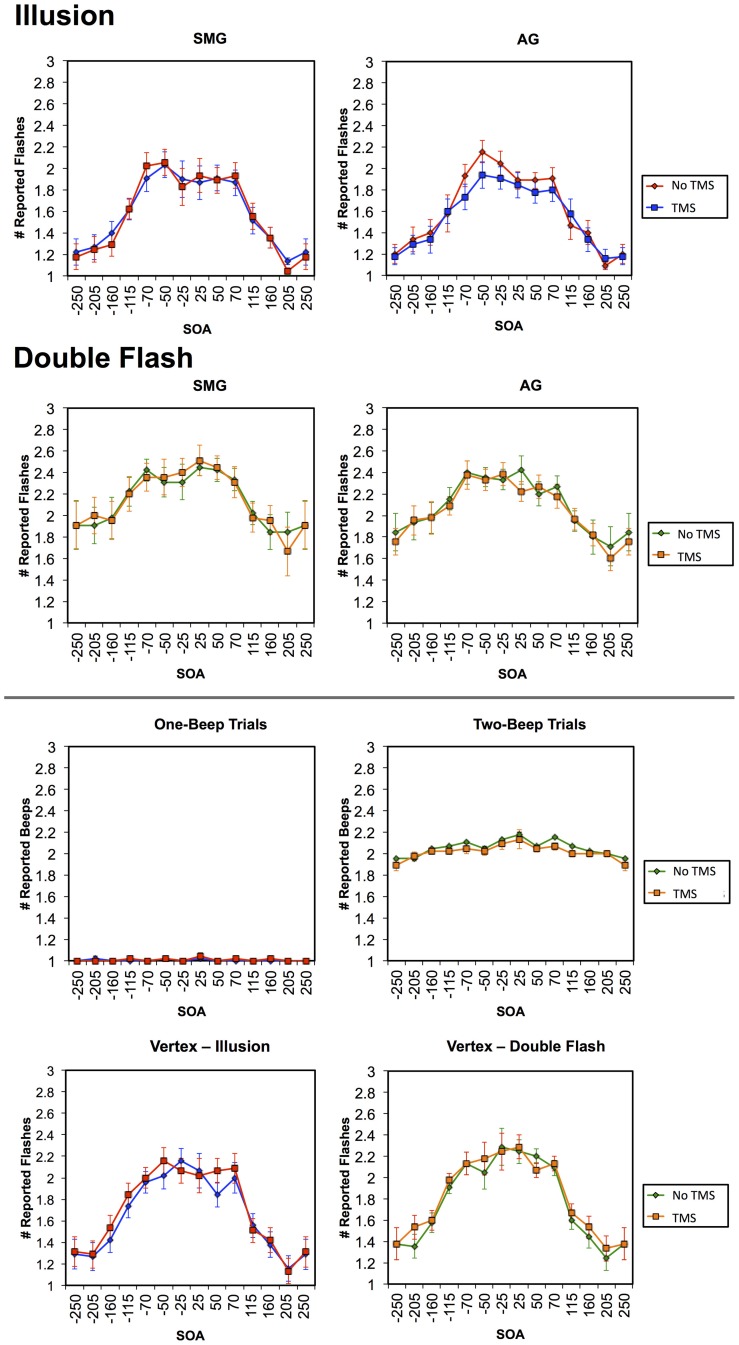
**Graphs in the first row represent the mean number of reported flashes during Illusion trials in TMS and no TMS conditions for stimulation of the SMG (left) and AG (right)**. Graphs in the second row represent the mean number of reported flashes during Double Flash trials in TMS and no TMS conditions for stimulation of the SMG (left) and AG (right). Figures in the third row represent the mean number of beeps reported in TMS and no TMS conditions for stimulation of the AG in an auditory control condition in which either one tone (left) or two tones (right) were presented. Figures in the bottom row represent the number of flashes in TMS and no TMS conditions involving Vertex stimulation for Illusion (left) and Double Flash (right) trials. Vertical bars represent standard error. Trials in which the accurate subject response was “one” (Illusion trials and one-beep auditory control trials) and are shown in blue (TMS) and red (no TMS); trials in which the accurate subject response was “two” (Double flash trials and two-beep auditory control trials) and are shown in orange (TMS) and green (no TMS).

**Figure 4:**
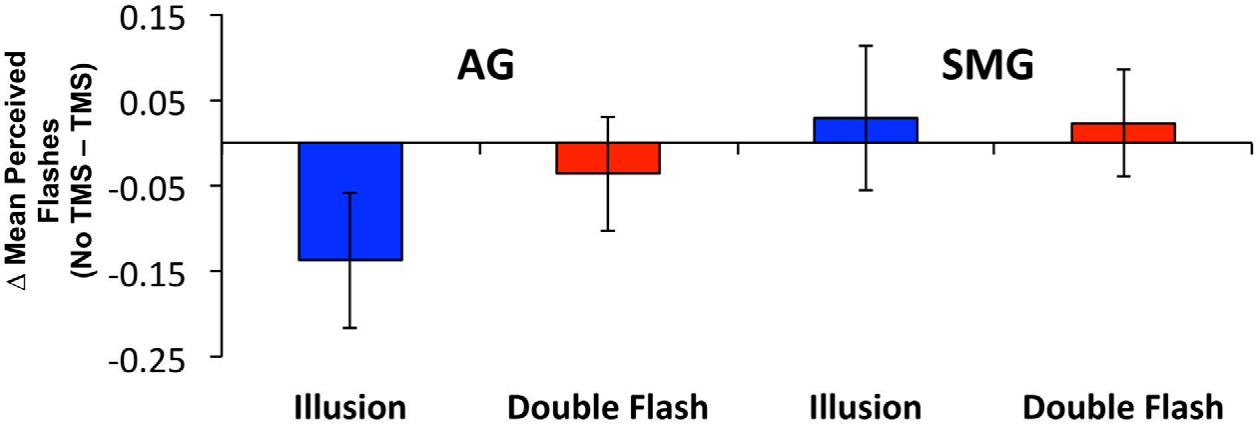
**The change in mean Peak Perceived Flashes (PPF) resulting from TMS by site (SMG, supramarginal gyrus; AG, angular gyrus), and trial type (Illusion, Double Flash)**. Blue bars represent Illusion trials; red bars represent Double Flash trials. Vertical bars represent standard error.

Multiple flashes were perceived in both the AG and SMG conditions on illusion trials. Multiple flashes were also perceived on double flash trials, although to a lesser extent than in illusion trials (Figure 3). A repeated measures ANOVA in which site (SMG, AG), TMS condition (no TMS, TMS), and trial type (illusion, double flash) were within subject factors revealed a significant main effect of trial type [*F*_(1, 8)_ = 13.655, *p* = 0.006] and a strong trend toward significant interaction between TMS condition and site [*F*_(1, 8)_ = 4.994, *p* = 0.056]. *Post-hoc t*-tests revealed a reduction of PPF after TMS but only for illusion trials and only after stimulation of the AG [one-sample *t*-test; *t*_(8)_ = 3.249, *p* = 0.002; *p*-values for all other conditions = 0.132). Moreover, the effect of TMS to the AG on PPF—as measured by the difference between the number of flashes perceived in the TMS and no TMS conditions—differed significantly from that observed after stimulation of the SMG during illusion trials [paired *t*-test; *t*_(8)_ = −2.429, *p* = 0.041], but not double flash trials [*t*_(8)_ = −1.045, *p* = 0.326] (Figure 4). Given that subjects were most likely to perceive the sound-induced flash illusion within the SOA window of −70 to +70 ms, we performed a binomial test comparing the direction of TMS effect for each subject at each SOA from −70 to 70 ms (54 total observations). This analysis demonstrated that the tendency to report fewer flashes after TMS was consistent across subjects during illusion trials after stimulation of the AG (*p* = 0.010). Three additional binomial tests showed no consistent direction of effect across subjects for double flash trials or for trails after stimulation of the SMG (*p* > 0.05).

We also reasoned that if TMS of the AG drives a more veridical perception of events during illusion trials, that there would be a shift toward reporting the presence of a single flash during illusion trials, and a shift away from reporting two or more flashes. Consistent with that notion, once again looking specifically at illusion trials within the SOA window of −70 to +70 ms, we found that subjects experienced a +44.7% increase in reporting one flash after receiving stimulation of the right AG compared to the pre-TMS condition and a −12.7% decrease in reporting 2 or more flashes.

Finally, on both the auditory and vertex control tasks there were no significant effects of TMS on the number of perceived tones either collapsed across all SOAs, within the −70 to 70 ms window, at any individual SOA (for all comparisons *p* > 0.05; Figure 3).

## Discussion

Consistent with the findings of Kamke et al. (2012), focal cortical inhibition in our subjects produced more veridical visual perception on the sound-induced flash illusion task when applied to the AG but not to the SMG of the right hemisphere. Moreover, the fact that TMS had no effect on performance in the double flash condition or in the auditory control condition suggests that the effect of TMS at AG is not attributable to a non-specific perturbation of auditory or visual perception. This finding argues that the AG of the right hemisphere is part of the neural network involved in audiovisual integration and suggests that this process at times sacrifices fidelity in the service of perceptual unity.

Phenomena such as the sound-induced flash illusion suggest the presence of a bias toward integrating auditory and visual information, even when these sensory streams are spatially or temporally incongruous. This notion is corroborated by other well-known audiovisual illusions. The ventriloquism effect, for example, is based on the ability to conjoin simultaneous but spatially separated auditory and visual information (De Gelder and Bertelson, 2003). With regard to time, it has been shown that when visual and auditory stimuli occur in close temporal proximity to each other, the perceived occurrence of each event is shifted toward temporal convergence (Fendrich and Corballis, 2001).

Extending this notion further, it is likely that imprecision in the integration of asynchronous multimodal events is an important feature of perceptual processing. Because information from various sensory modalities propagates at different rates in the environment and follows different pathways in the nervous system, it is likely that some tolerance of asynchrony permits perceptual integration of stimuli that are of common origin in the world, but mismatched in neural processing (e.g., Slutsky and Recanzone, 2001; Lewald and Gusky, 2003). While this perceptual strategy leads to the highest fidelity judgments about multisensory events on average, illusions like the one employed in this study reveal rare exceptions in which this process fails to accord with actual auditory and visual events. Our observation that cortical inhibition interferes with this kind of illusion implies that tolerance for multisensory asynchrony is actively mediated, and is not a passive reflection of limited perceptual acuity.

Converging lines of evidence provide insights into the neural mechanisms of audiovisual integration. In animals, it has been shown that audiovisual stimuli originating from a single event evoke super-additive responses in a network of brain regions that includes the superior colliculus, and frontal, parietal, and temporal association cortices (Stein, 1998; Fuster et al., 2000). Functional imaging studies in humans have implicated similar brain regions in multimodal integration (e.g., Calvert, 2001), and have demonstrated that some audiovisual illusions specifically engage these regions (Bushara et al., 2003). These studies consistently point to the RIPL as part of the network of association cortices engaged in multimodal integration. Our and Kamke et al.'s findings extend observations from functional imaging studies in two important respects. First, TMS-induced behavioral changes allow for causal inferences regarding neural structure-function relationships that cannot be made using fMRI. Second, our data fractionate the inferior parietal lobule anatomically in a way not previously observed in animal studies or neuroimaging; TMS to the AG but not the nearby SMG is associated with a significant change in performance.

The inferior parietal lobule has been implicated in a variety of mental operations in addition to audiovisual integration, including quantity estimation (e.g., Dehaene et al., 2003). One alternative account of the current results is that TMS of the right AG might have resulted in a transient manipulation of quantity judgments. While this could conceivably account for a transient change in the enumeration of multiple transient sensory events, it does not adequately explain why the effect of TMS would be more pronounced in single flash illusion trials than in double flash trials or why the TMS effect would be restricted to only audiovisual trials.

Other investigations into the neural basis of audiovisual integration indicate that basic elements of audiovisual integration may be processed at the level of primary sensory cortices, and that activity in the primary sensory cortex of one modality may directly impact activity in the other (e.g., Martuzzi et al., 2007; Raij et al., 2010; Romei et al., 2012). Several have employed the same sound-induced flash illusion used in the current study (e.g., Shams et al., 2001; Arden et al., 2003; de Haas et al., 2012). Shams et al. (2005) used magnetoencephalography to demonstrate early modulation of activity in the occipital lobe (35–65 ms from the onset of the visual stimulus) during perception of the sound-induced flash illusion. Association cortices were engaged later (~150 ms post-stimulus), when modulation of activity in occipital, parietal, and anterior areas was observed. Crucially, Shams and colleagues observed that, when subjects did not perceive the illusion, there were no changes in occipital or parietal activity. Mishra et al. (2007) similarly used event-related potentials to report early modulation of visual cortical activity (30–60 ms post-stimulus) and later modulation of polymodal cortex (the superior temporal gyrus in their investigations; ~150 ms post-stimulus), and found that activity in the latter region was modulated by whether or not the illusion was perceived. Of note, other investigators have reported functional imaging evidence suggesting that primary visual cortex activation may correlate with perception of this illusion (e.g., Watkins et al., 2006, 2007). The notion that association areas like the AG are critically involved in multisensory integration is further supported by the observation that the application of TMS to this site interferes with tasks that rely on crossmodal information transfer, such as intersensory cuing of attention (Chambers et al., 2007).

We incidentally note an apparent asymmetry between negative and positive SOAs in illusion trials; illusory flashes appear to be perceived more frequently when a second beep occurs after the flash than before. Similar findings have been observed previously in the literature, and it is thought that the presence of the asymmetry is due to differences in the physical and neural transmission times for auditory and visual signals. For instance, Fujisaki et al. (2004) noted this when they tested whether presenting audiovisual information in an asynchronous manner could alter participants' performance on a multimodal simultaneity judgment task. The asymmetry is noted in other studies as well (e.g., Zampini et al., 2005; Powers et al., 2009).

One interesting potential interpretation of our findings derives form prior work by Shams and colleagues (2005) indicating that human performance on the sound-induced flash illusion closely mirrors that of an ideal observer that follows Bayesian rules of conditional probability to determine when and how to combine auditory and visual signals (Shams et al., 2005). From this perspective, we can posit that disrupting AG function with TMS may cause subjects to temporarily rely less on Bayesian integration of prior sensory events in their subsequent interpretation of new sensory input, leading to perceptual outcomes that are statistically less optimal, but are more veridical in the context of this specific visual illusion.

Interestingly, the mean number of reported flashes on double flash trials exceeded the number presented, suggesting some degree of additional illusory perception even in trials wherein the number of auditory and visual stimuli were matched. However, the magnitude of this illusory effect, as estimated by the difference between the number of flashes reported and the number presented, was larger for single flash illusion trials (~1 additional reported flash within the −70 to 70 ms window) than for double flash trials (~0.4 flashes). We believe that stimulation of the AG may have also resulted in a decrease in flash perception during double flash trials, but to a lesser extent than in single flash trials because the illusion effect was smaller (Figure 3). In addition, this possible TMS effect in the double flash condition is not present at larger SOAs, where the number of presented and perceived flashes is two. In this SOA range TMS of the AG does not result in a non-specific reduction in perception of multiple flashes, implying that the TMS effect is not due to a non-specific disruption of visual processing or to an overall perturbation of magnitude judgment.

One limitation in this study was the relatively small number of stimuli at each SOA in each condition; a necessary constraint due to the relatively brief duration of anticipated TMS effects. A second potential limitation of the study is that the double-flash control condition could have been designed differently, so as more thoroughly avoid the percept of illusory flashes in this control task. Finally, because this study was limited to the right AG and SMG, the role of the homologous regions on the left hemisphere remains unknown. Nonetheless, our findings independently confirm recent evidence that that disruption of the right AG perturbs an illusory effect that depends on the interaction between audition and vision, and imply that this region is part of a network of brain areas that actively mediates the ability to create conjunctions between auditory and visual events, whether real or illusory.

### Conflict of interest statement

The authors declare that the research was conducted in the absence of any commercial or financial relationships that could be construed as a potential conflict of interest.
